# Supplementary material for: Optimization of the cytogenetic protocol for Pangasianodon hypophthalmus (Sauvage, 1878) and Clarias gariepinus (Burchell, 1822)
Source: PeerJ. 2018 Nov 5;6:e5712. doi: 10.7717/peerj.5712 (PMC6223236; doi:10.7717/peerj.5712)
Supplement: Supplemental Information 2 — Cytogenetic metaphase spread with the different treatments. [file peerj-06-5712-s002.pdf]

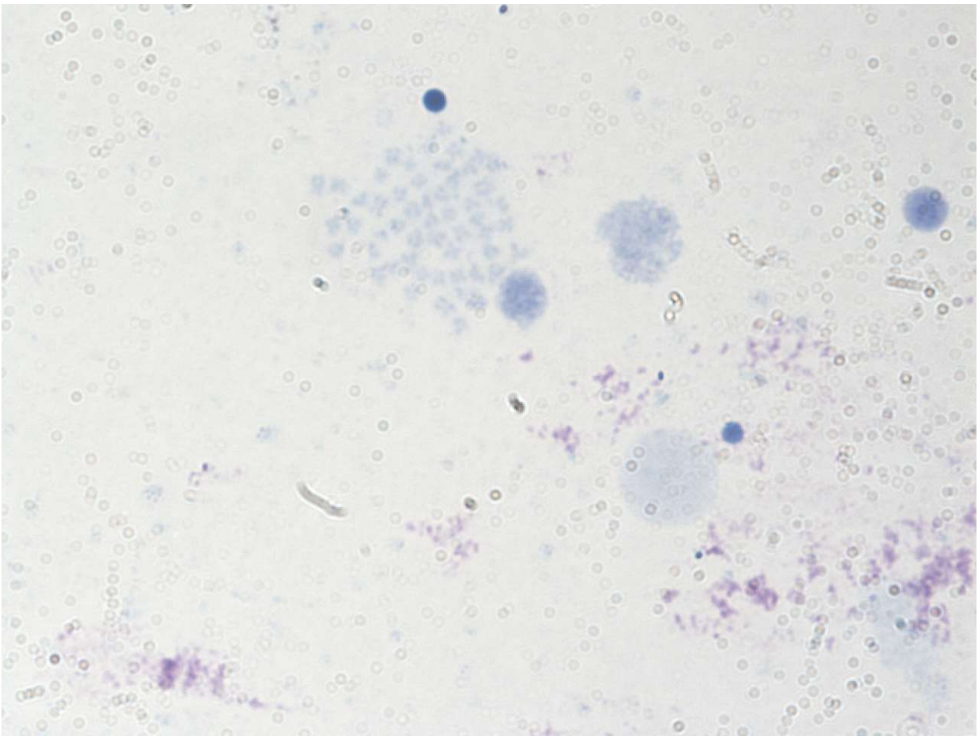

Sample of unprocessed metaphase spread from first treatment

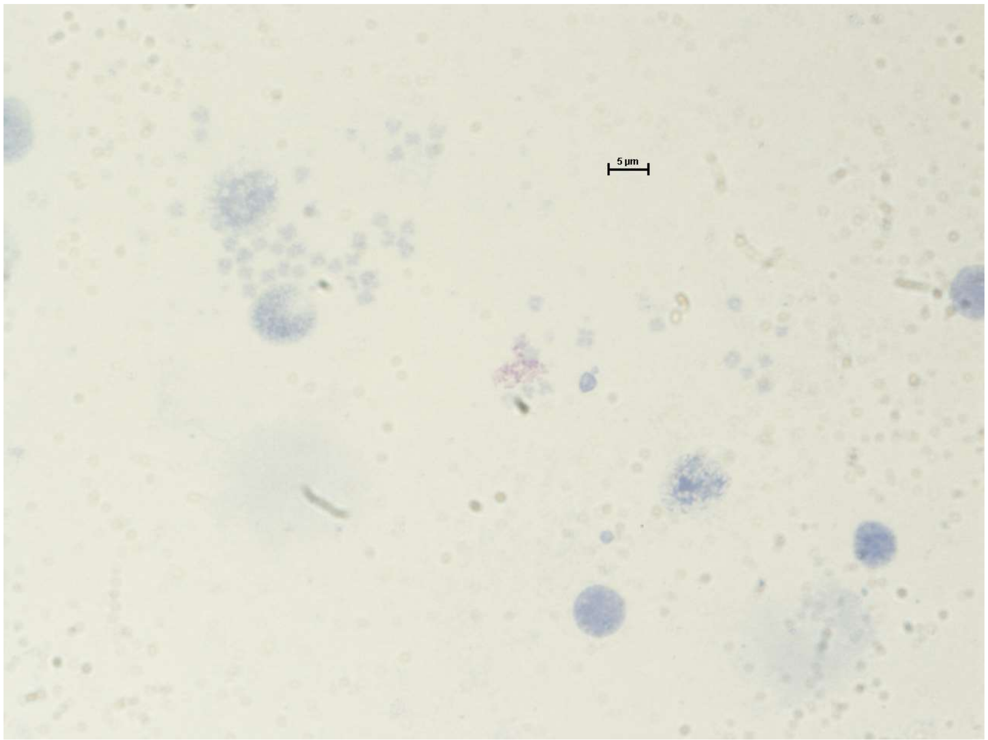

Sample of unprocessed metaphase spread from first treatment

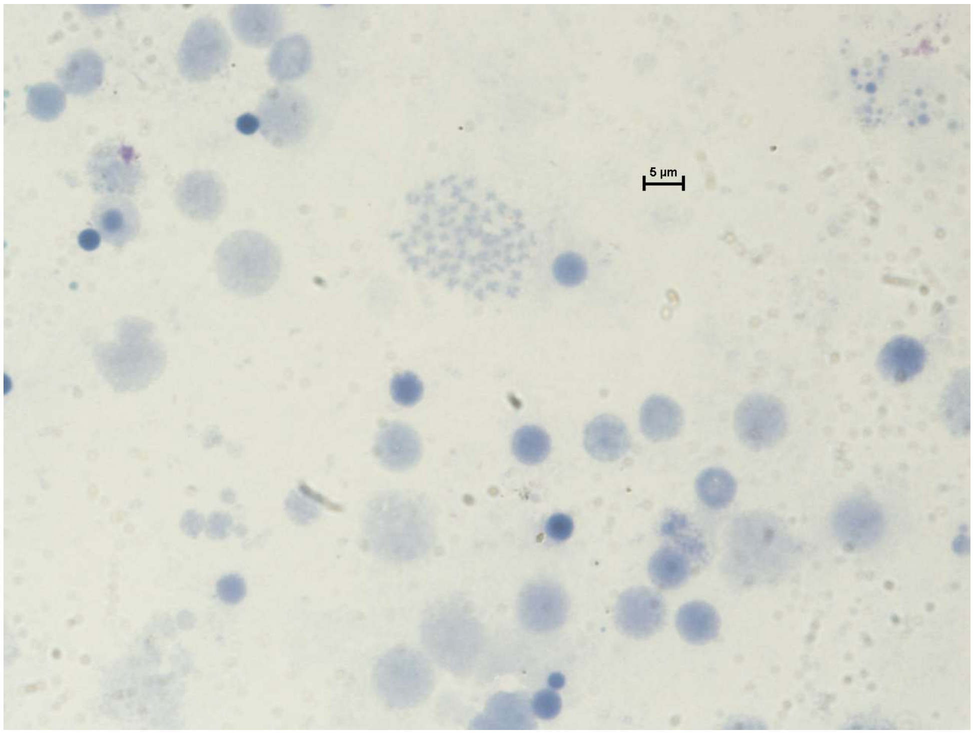

Sample of unprocessed metaphase spread from first treatment

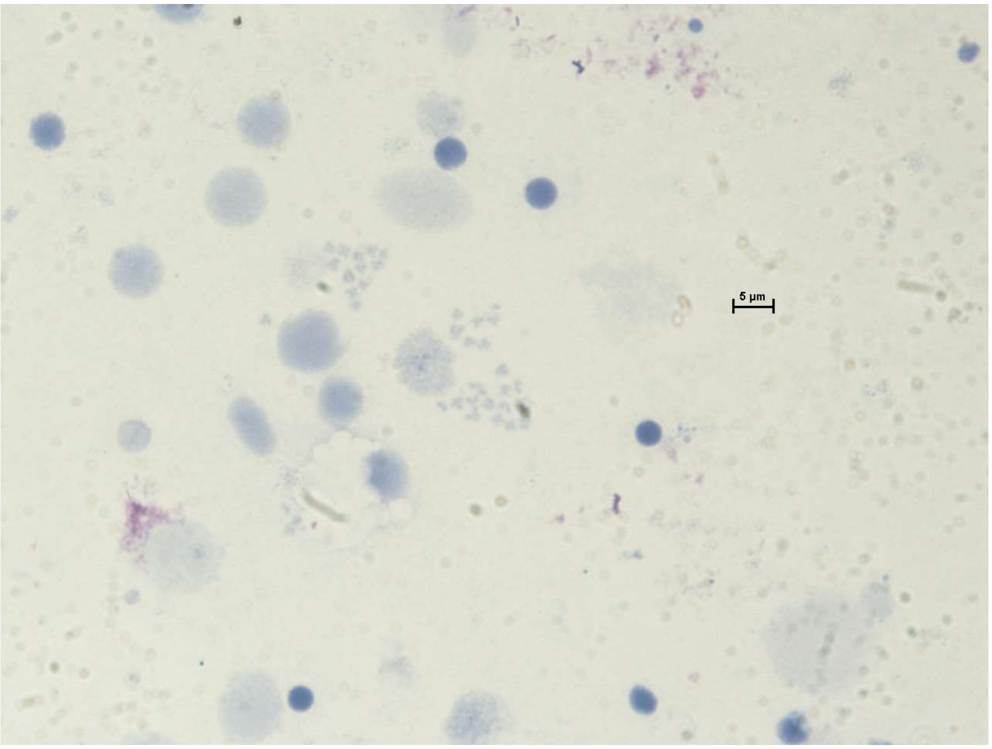

Sample of unprocessed metaphase spread from first treatment

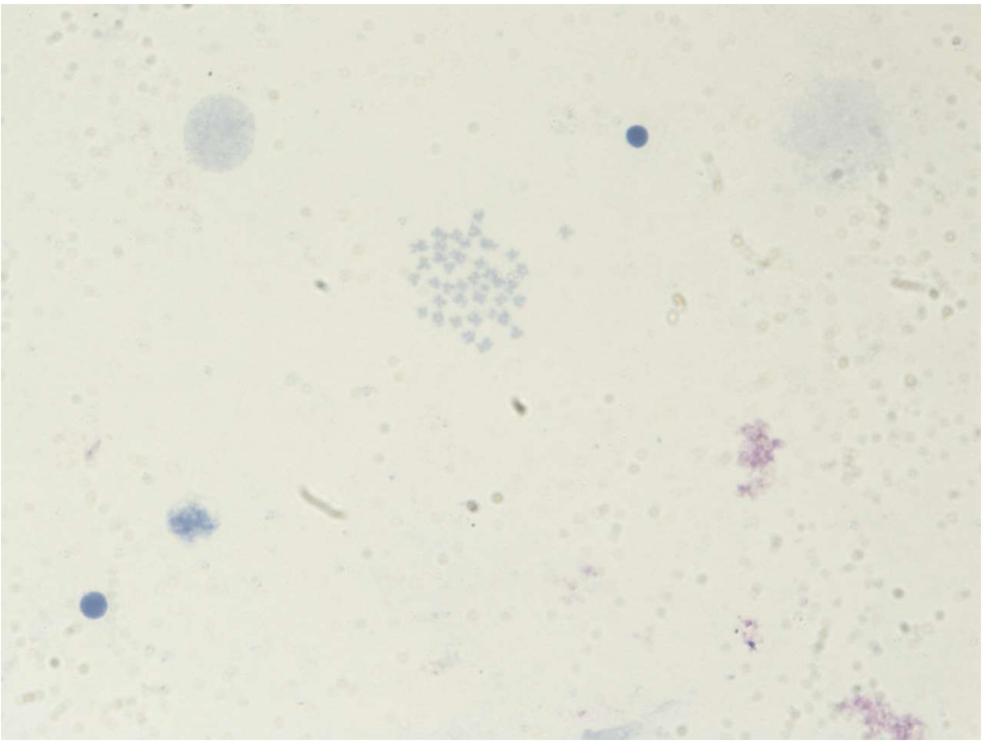

Sample of unprocessed metaphase spread from first treatment

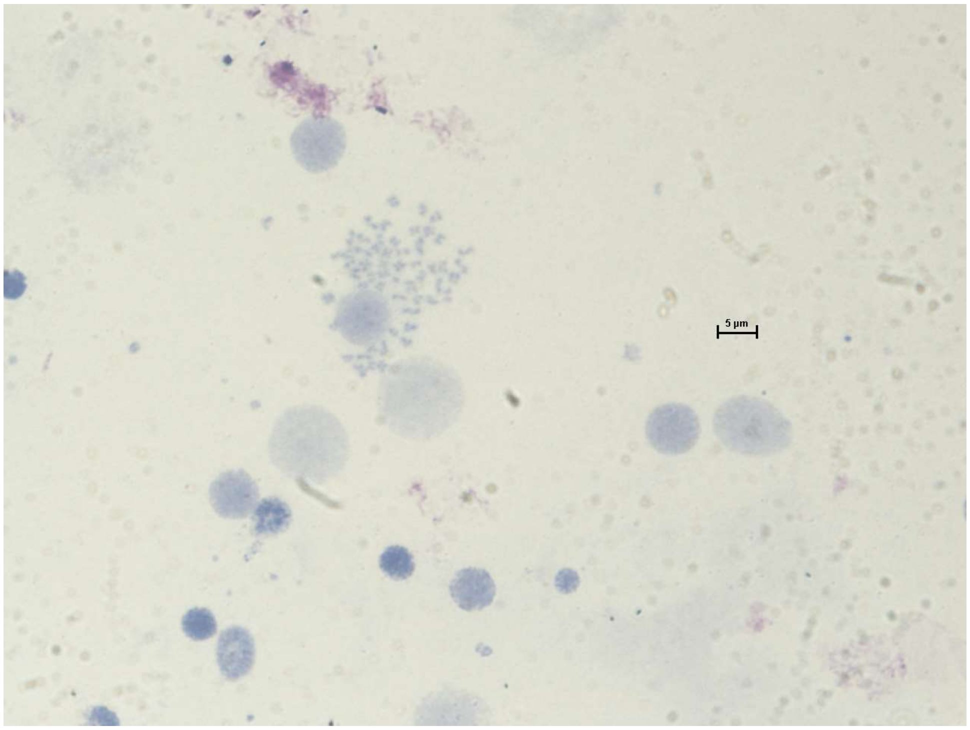

Sample of unprocessed metaphase spread from first treatment

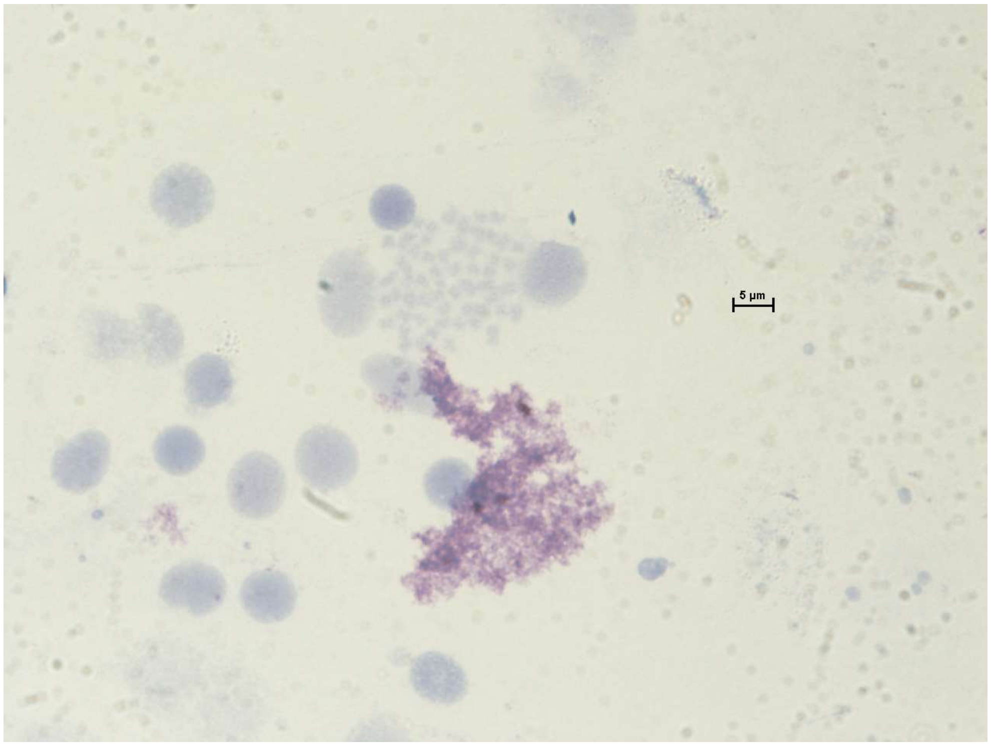

Sample of unprocessed metaphase spread from second treatment

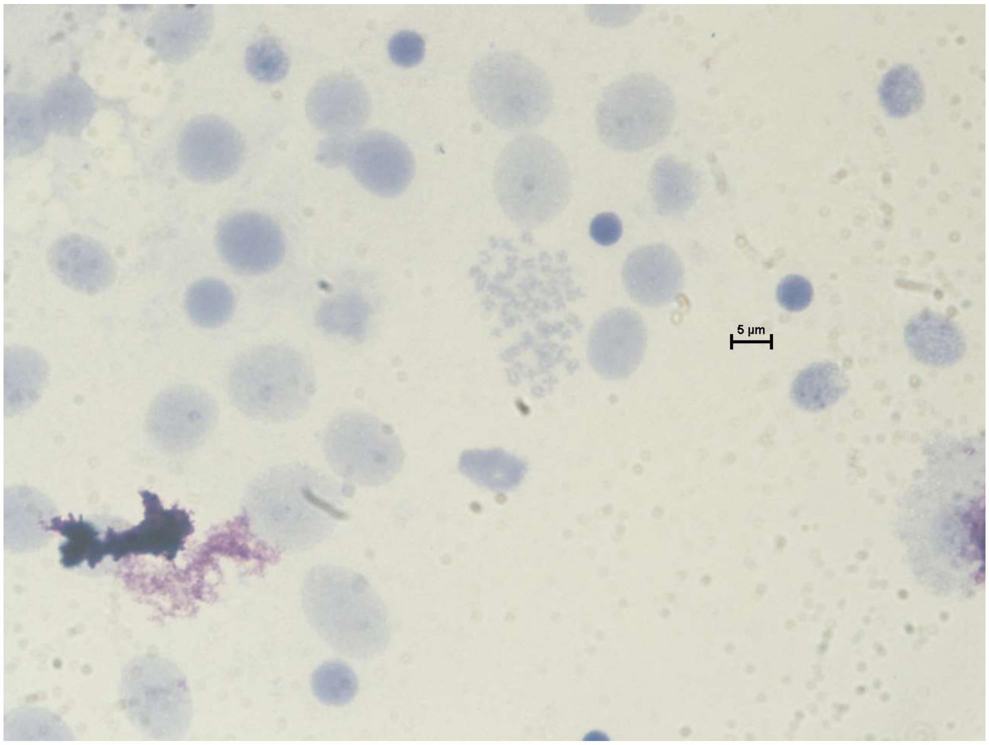

Sample of unprocessed metaphase spread from second treatment

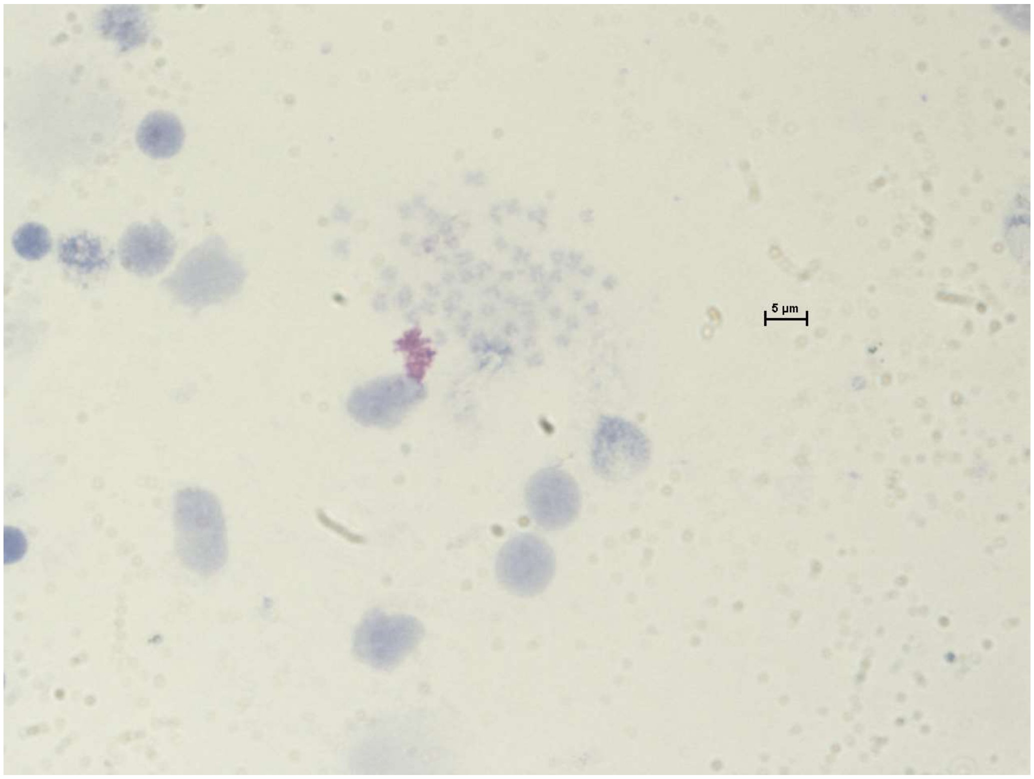

Sample of unprocessed metaphase spread from second treatment

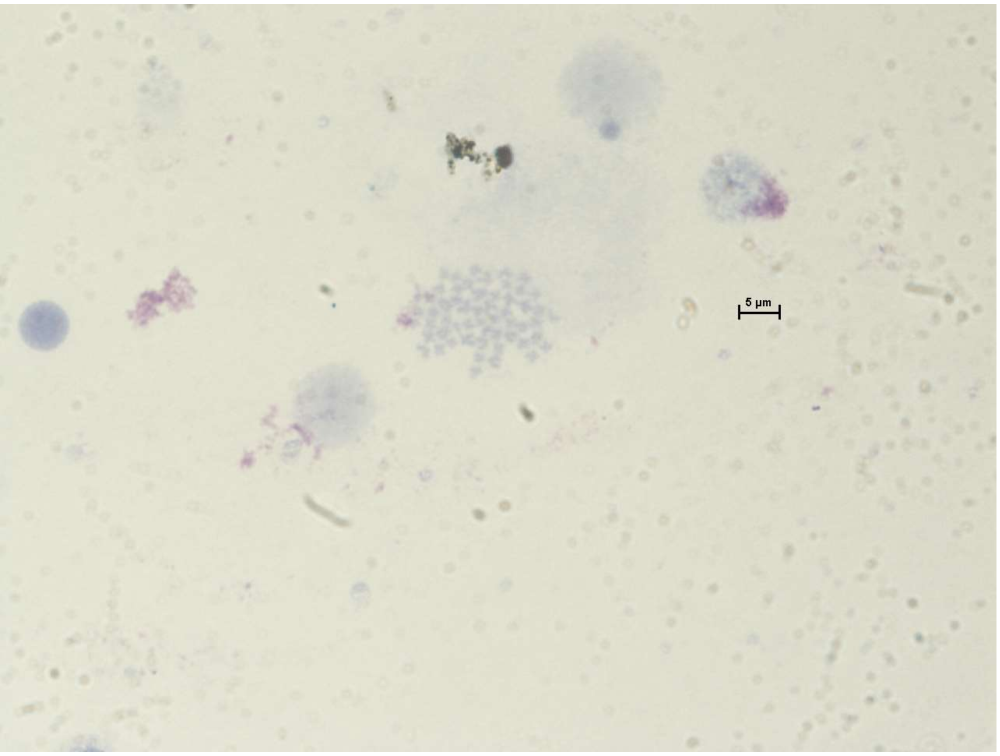

Sample of unprocessed metaphase spread from second treatment

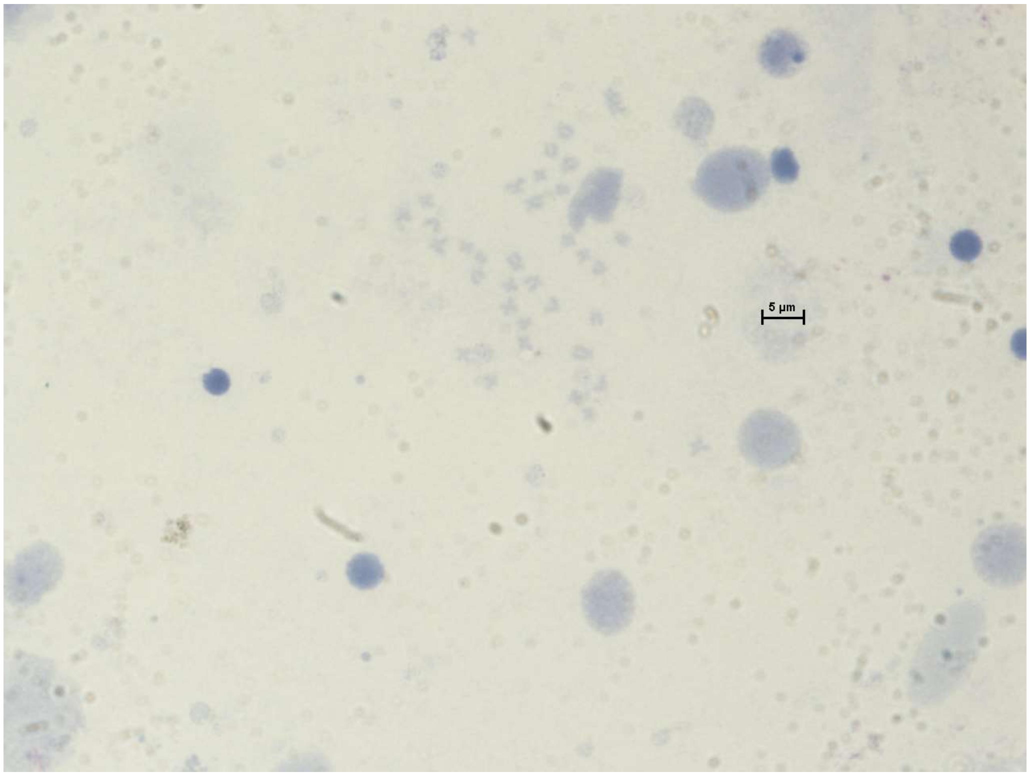

Sample of unprocessed metaphase spread from second treatment

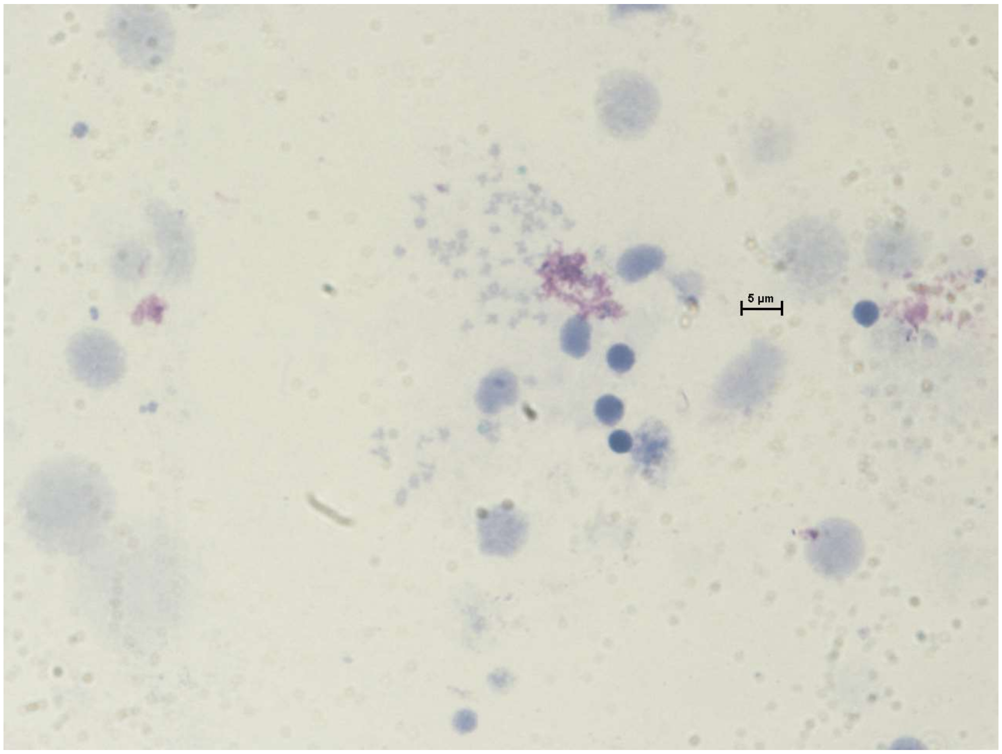

Sample of unprocessed metaphase spread from second treatment

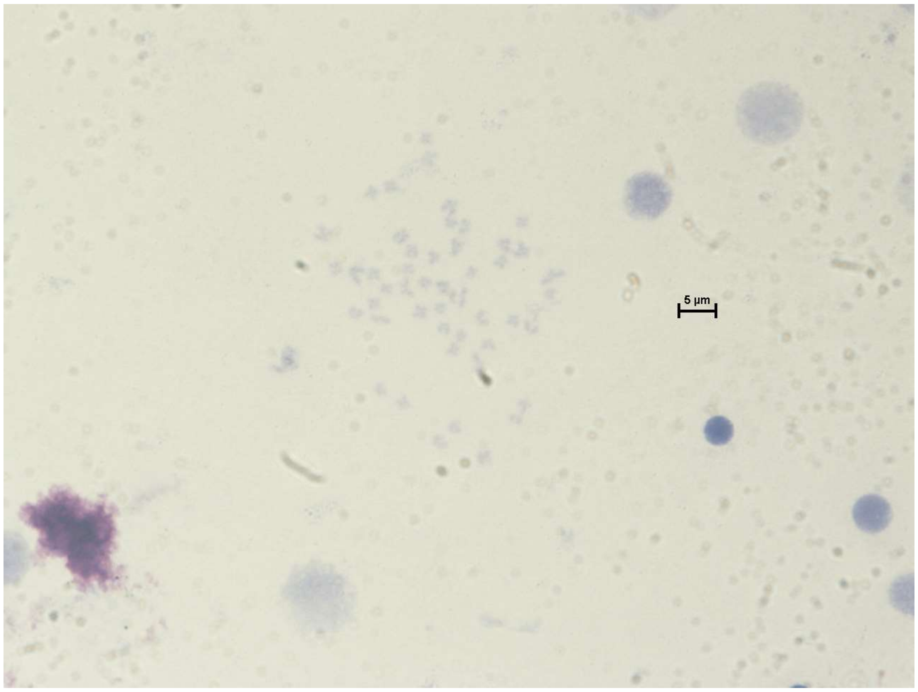

Sample of unprocessed metaphase spread from third treatment

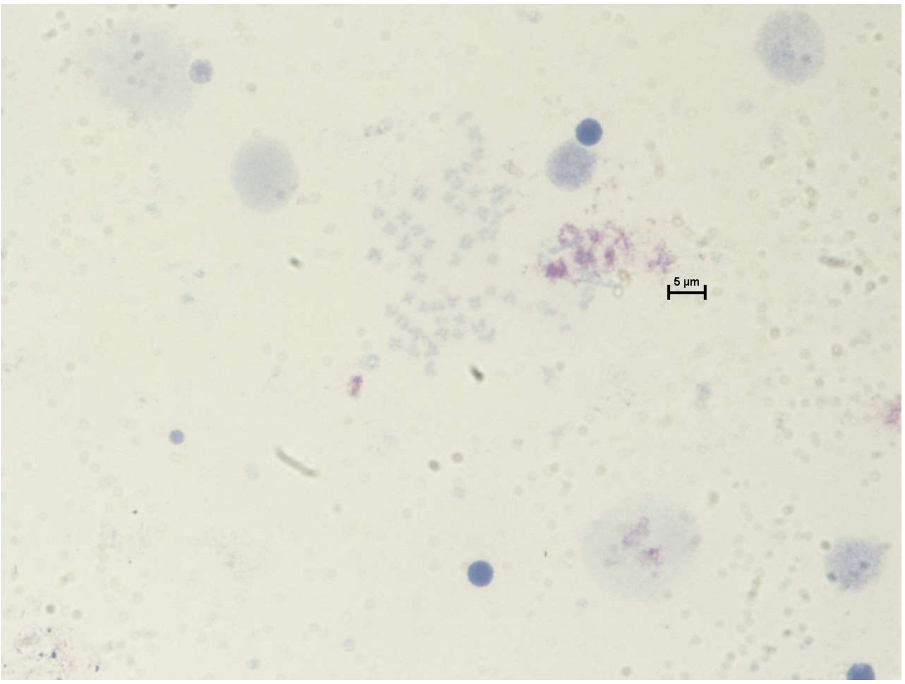

Sample of unprocessed metaphase spread from third treatment

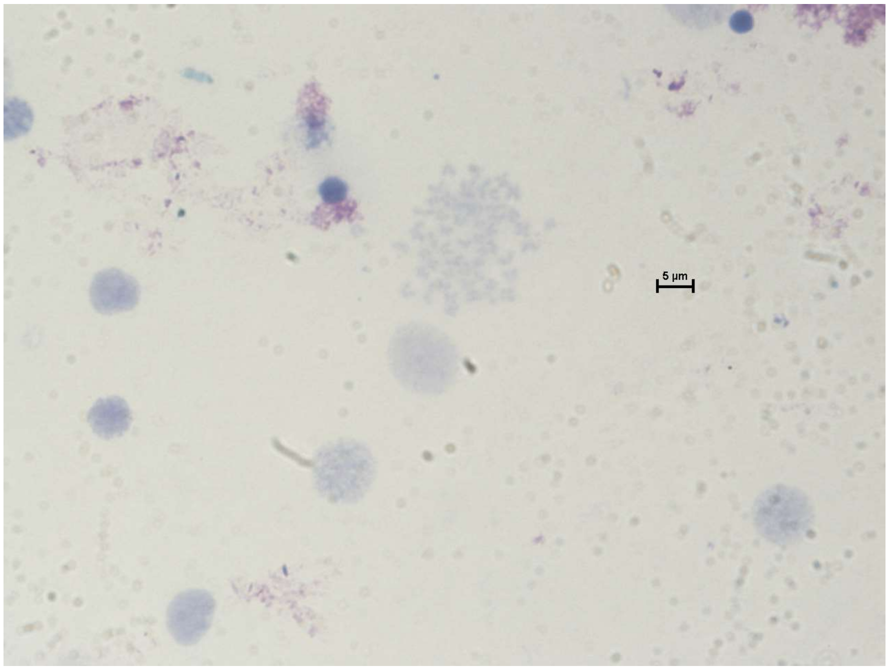

Sample of unprocessed metaphase spread from third treatment

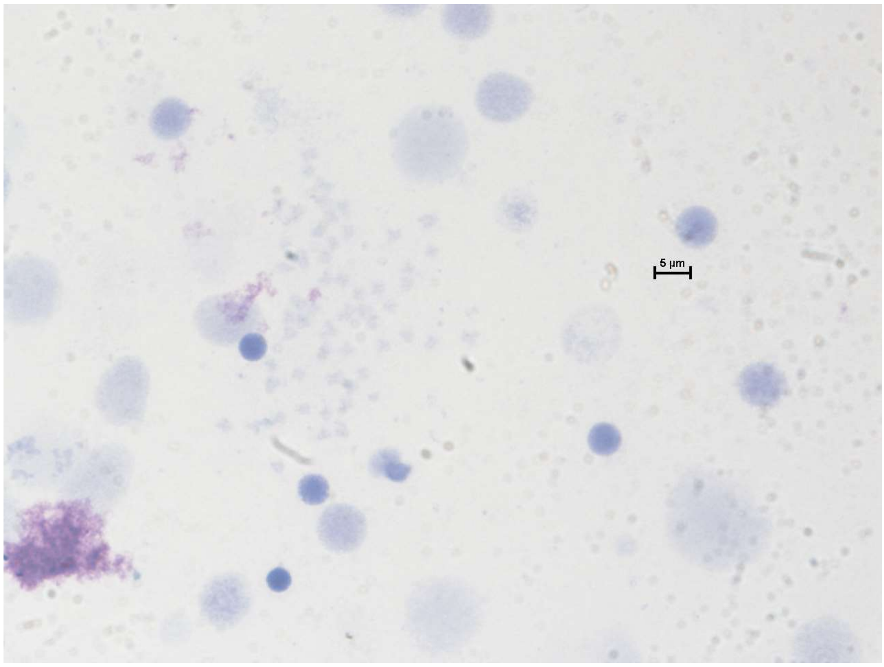

Sample of unprocessed metaphase spread from third treatment

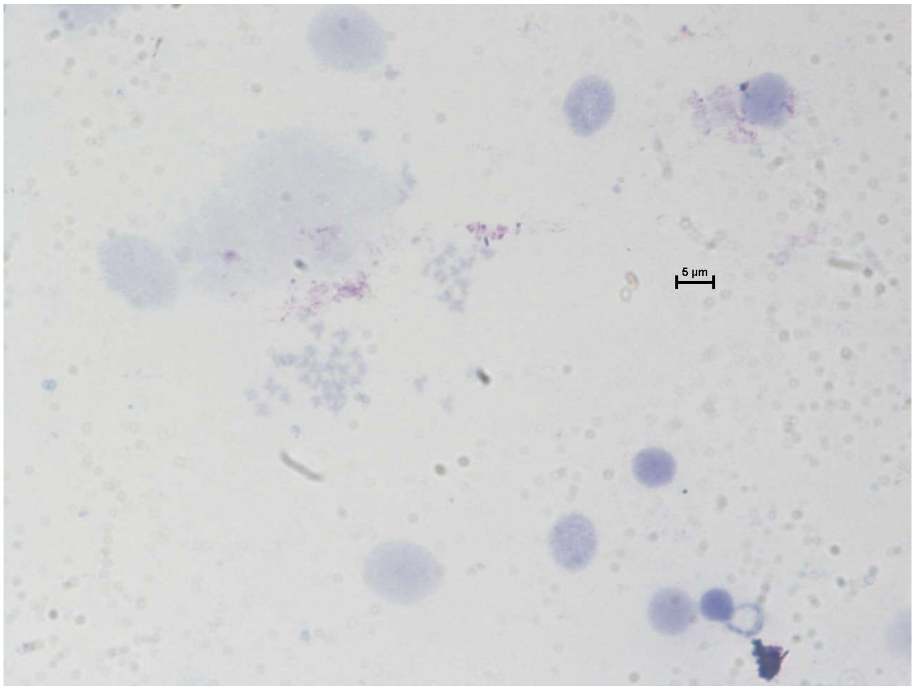

Sample of unprocessed metaphase spread from third treatment

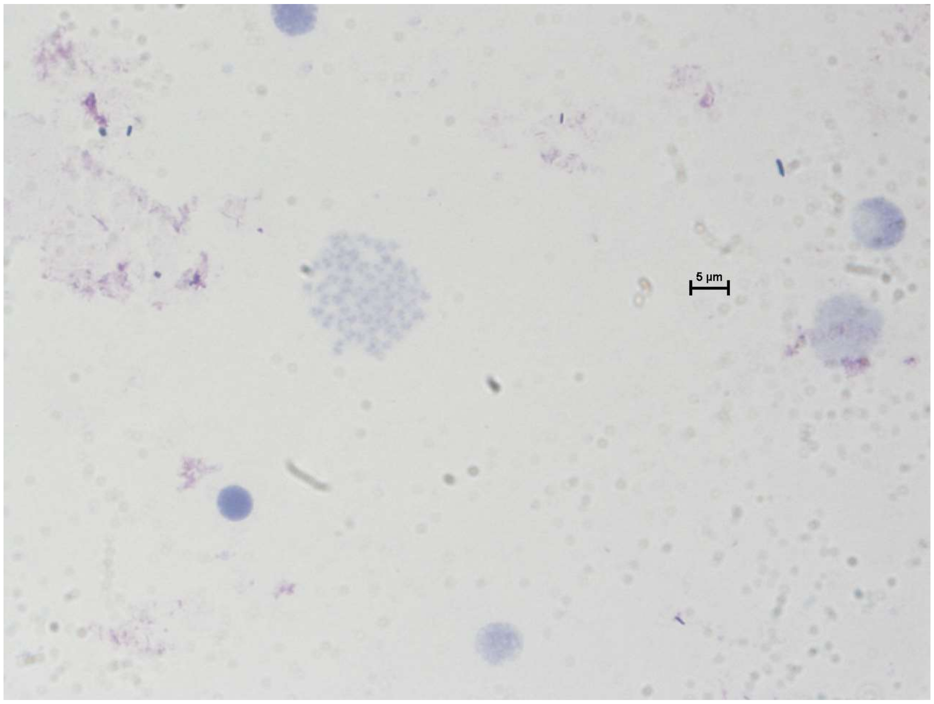

Sample of unprocessed metaphase spread from third treatment

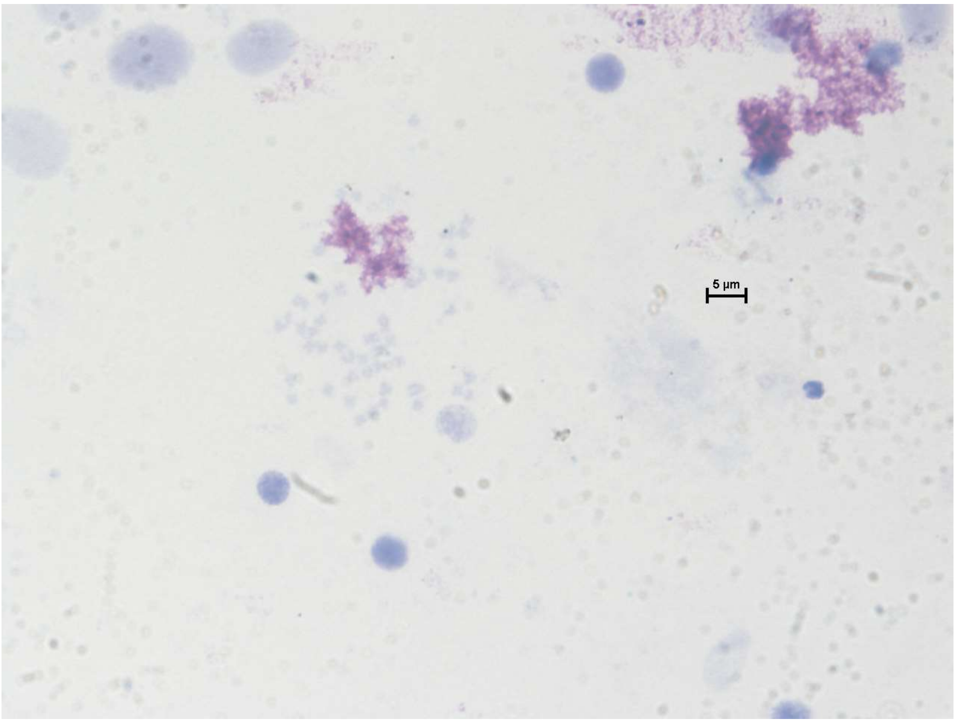

Sample of unprocessed metaphase spread from fourth treatment

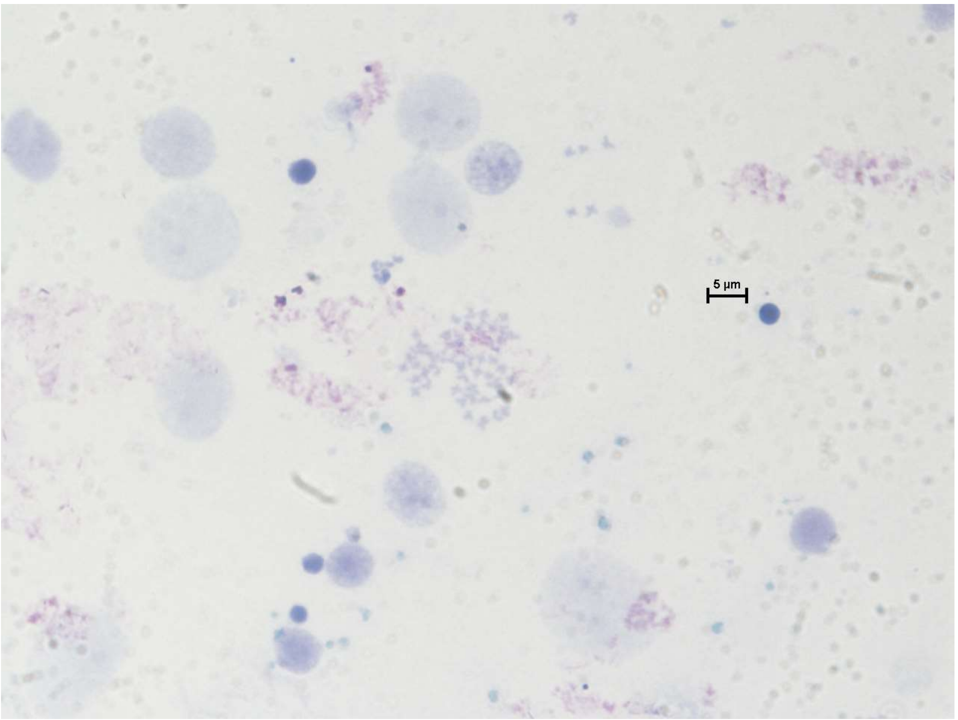

Sample of unprocessed metaphase spread from fourth treatment

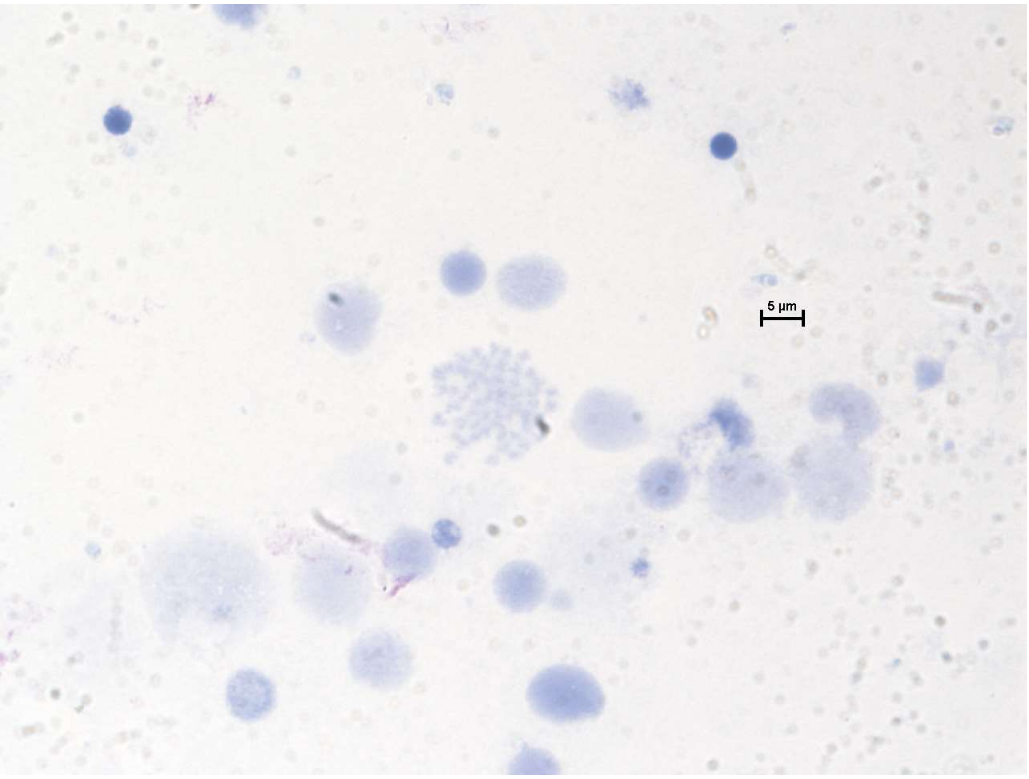

Sample of unprocessed metaphase spread from fourth treatment

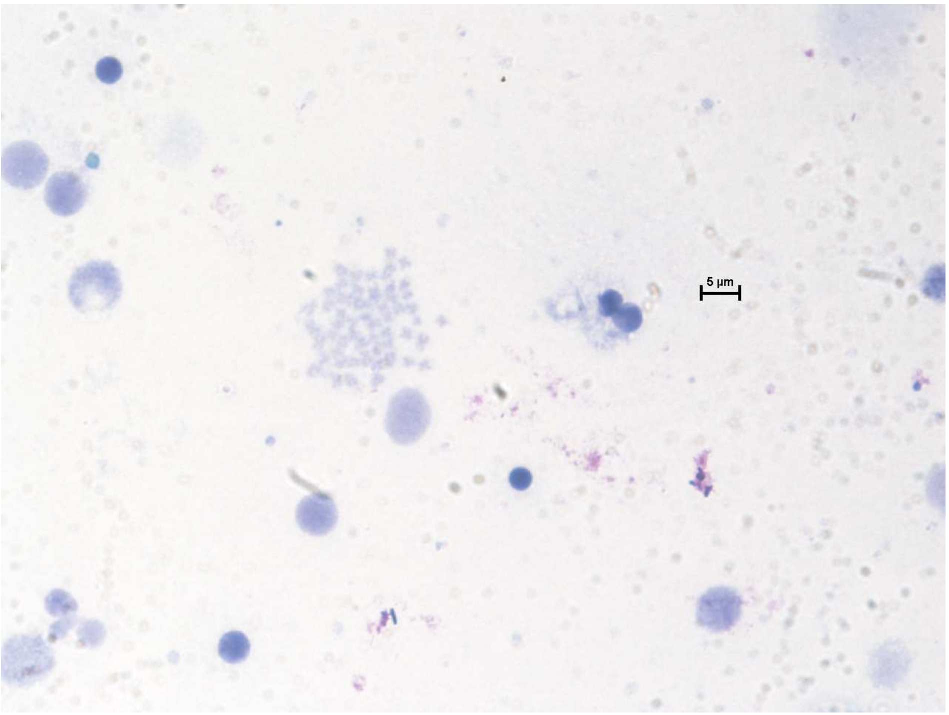

Sample of unprocessed metaphase spread from fourth treatment

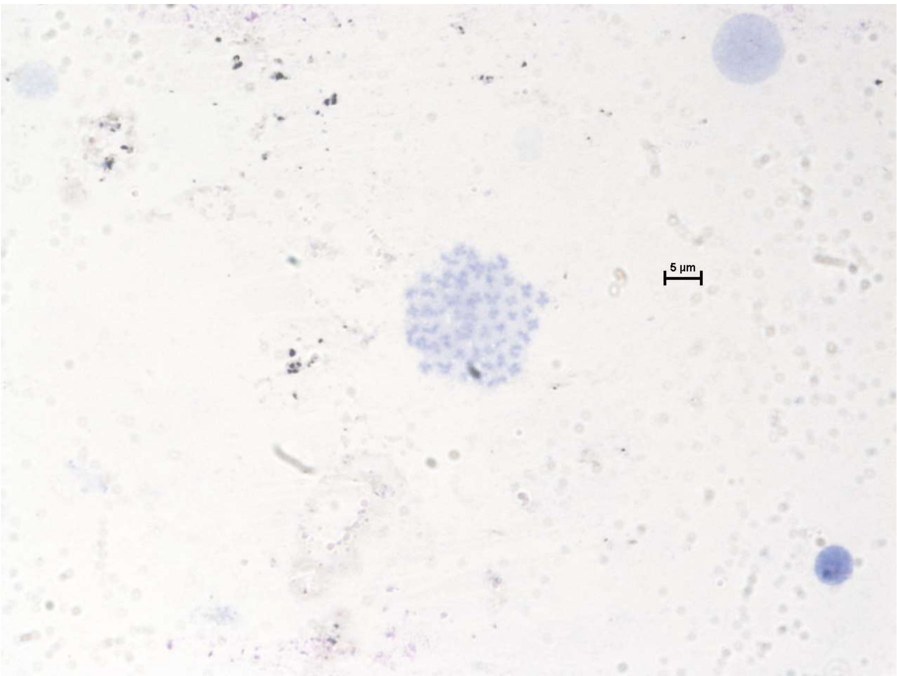

Sample of unprocessed metaphase spread from fourth treatment

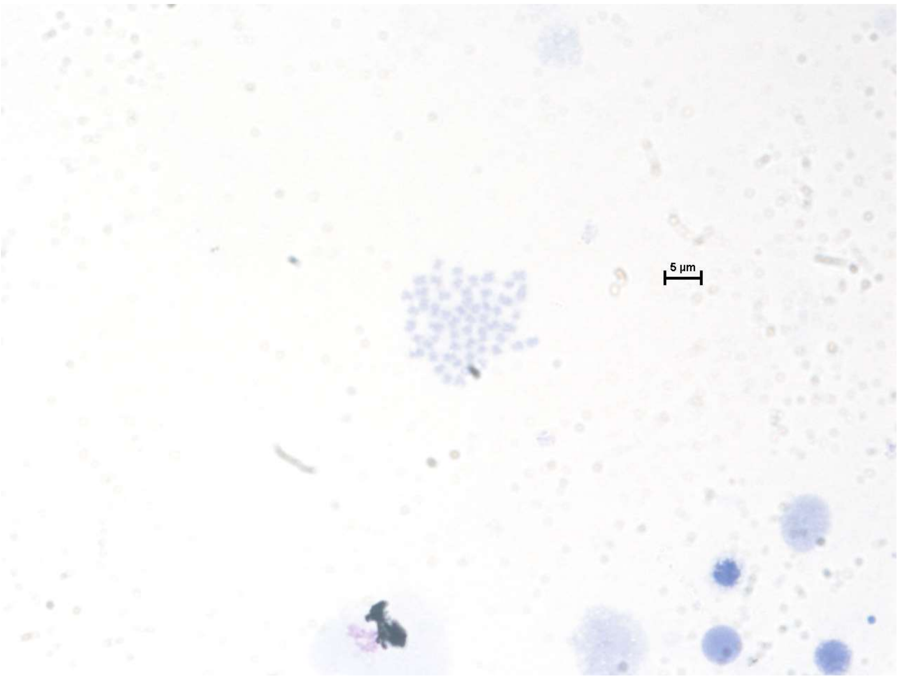

Sample of unprocessed metaphase spread from fourth treatment
